# Investigation of Mechanical Properties and Salt Spray Corrosion Test Parameters Optimization for AA8079 with Reinforcement of TiN + ZrO_2_

**DOI:** 10.3390/ma14185260

**Published:** 2021-09-13

**Authors:** T. Sathish, V. Mohanavel, T. Arunkumar, T. Raja, Ahmad Rashedi, Ibrahim M. Alarifi, Irfan Anjum Badruddin, Ali Algahtani, Asif Afzal

**Affiliations:** 1 Department of Mechanical Engineering, Saveetha School of Engineering, SIMATS, Chennai 602105, Tamil Nadu, India; 2 Centre for Materials Engineering and Regenerative Medicine, Bharath Institute of Higher Education and Research, Chennai 600073, Tamil Nadu, India; mohanavel2k16@gmail.com; 3 Department of Mechanical Engineering, CMR Institute of Technology, Bengaluru 560037, Karnataka, India; arunmailinbox@gmail.com; 4 Department of Mechanical Engineering, Vel Tech Rangarajan Dr. Sagunthala R&D Institute of Science and Technology, 400 Feet Outer Ring Road, Avadi, Chennai 600062, Tamil Nadu, India; rajasd28@gmail.com; 5 School of Mechanical & Aerospace Engineering, Nanyang Technological University, 50 Nanyang Avenue, Singapore 639798, Singapore; amma0002@e.ntu.edu.sg; 6 Department of Mechanical and Industrial Engineering, College of Engineering, Majmaah University, Al-Majmaah, Riyadh 11952, Saudi Arabia; i.alarifi@mu.edu.sa; 7 Engineering and Applied Science Research Center, Majmaah University, Al-Majmaah, Riyadh 11952, Saudi Arabia; 8 Research Center for Advanced Materials Science (RCAMS), King Khalid University, P.O. Box 9004, Abha 61413, Saudi Arabia; irfan@kku.edu.sa (I.A.B.); alialgahtani@kku.edu.sa (A.A.); 9 Mechanical Engineering Department, College of Engineering, King Khalid University, Abha 61411, Saudi Arabia; 10 Department of Mechanical Engineering, P. A. College of Engineering, Affiliated to Visvesvaraya Technological University, Belagavi, Mangaluru 574153, Karnataka, India; 11 Department of Mechanical Engineering, School of Technology, Glocal University, Delhi-Yamunotri Marg, SH-57, Mirzapur Pole, Saharanpur 247121, Uttar Pradesh, India

**Keywords:** ultimate tensile strength, wear, corrosion, reinforcement, titanium nitride, pH value, salt spray, ANOVA

## Abstract

This work mainly focuses on increasing the mechanical strength and improving the corrosion resistance of an aluminum alloy hybrid matrix. The composites are prepared by the stir casting procedure. For this work, aluminum alloy 8079 is considered as a base material and titanium nitride and zirconium dioxide are utilized as reinforcement particles. Mechanical tests, such as the ultimate tensile strength, wear, salt spray corrosion test and microhardness test, are conducted effectively in the fabricated AA8079/TiN + ZrO_2_ composites. L9 OA statistical analysis is executed to optimize the process parameters of the mechanical and corrosion tests. ANOVA analysis defines the contribution and influence of each parameter. In the tensile and wear test, parameters are chosen as % of reinforcement (3%, 6% and 9%), stirring speed (500, 550 and 600 rpm) and stirring time (20, 25 and 30 min). Similarly, in the salt spray test and microhardness test, the selected parameters are: percentage of reinforcement (3%, 6% and 9%), pH value (3, 6 and 9), and hang time (24, 48 and 72 h). The percentage of reinforcement highly influenced the wear and microhardness test, while the stirring time parameter extremely influenced the ultimate tensile strength. From the corrosion test, the hang time influences the corrosion rate. The SEM analysis highly reveals the bonding of each reinforcement particle to the base material.

## 1. Introduction

The stir casting process is a reliable process and it is used frequently for ferrous and nonferrous components. The stir casting process provides the homogeny, durability and strength of the mixed composite after it forms the solid shape. The powder is mixed well in the base materials to make up a rigid solid structure after solidification. The stirring parameters, such as stirring speed, stirring time and stirring temperature, are highly involved in altering the strength of the hybrid composites [1].

Additionally, the aluminum alloy 6061 hybrid MMCs (Metal matrix composites) with reinforcement of silicon carbide uses a banana and jute to increase the strength of the composites [2]. The authors conducted the tensile test, hardness test and percentage of elongation. 

Using different reinforcement percentages, the banana addition highly enhanced the tensile strength, hardness and percentage elongation. Researchers have reported that, in different layer sequences, excellent wear resistant was offered by a jute–rubber–jute composite for varying wear parameters [3].

Aluminum metal matrix composites are prepared through the stir casting method; authors in [4] used as base material of aluminum with 4.5% copper alloy [4]. In their investigation, bamboo leaf ash is used as the the reinforcement material with different percentage levels, e.g., 2%, 4% and 6%. The obtained results showed that by increasing the bamboo leaf ash percentage, the density of the composite decreased and the porosity increased. Better tensile strength and hardness were found by use of 4% reinforcement, while continually increasing the percentage level of the bamboo leaf ash reduced the tensile strength and hardness of the composites.

Use of the stir casting process was conducted effectively to produce AMCs with reinforcement of titanium diboride [5]. The authors studied the mechanical strength, and fracture influences and microstructure (OM and SEM) analyses of the tensile samples proved that the prepared composites offered excellent bonding between the base material and titanium diboride. Increasing the percentage of titanium diboride reinforced particles improved and increased the tensile strength. It can be observed from the SEM analysis that the growth of nucleation and small voids induced the fractures in the tensile test samples. 

A study has suggested and that the reinforcement of boron carbide particles offered good mechanical properties for the aluminum alloy [6]; for their investigation, different percentage levels of boron carbide particles were reinforced into aluminum alloy AA6061 during the stir casting process. The average particle size (25 μm) of the boron carbide induced the highest mechanical strength. Higher boron carbide percentage levels increased the tensile strength and hardness of the aluminum metal matrix composites.

Agitation process (stirring) is one of the predominant factors in the stir casting method to increase the strength of the composites. At the secondary level of the stir casting, the stirring time had a major role in modifying the strength and structure of the composites [7]. Finally, the stir casting temperature was used to increase the melting point of both base materials as well as the reinforced particles.

This parameter acknowledges the uniform mixture obtained in the prepared composites. The stir casting parameters, such as stirring speed, stirring time and stirring temperature, were influenced to amend the strength of the casted samples [8]. Stirring speed highly influenced the obtainment of the homogeneous mixture of the composites, and the stirring time increased the strength of the composites due to the longer duration resulting in the reinforced particles being well settled into the base materials [9].

The stir casting temperature stimulated the high melting point of the composites, taking the minimum time to blend the particles in a good fashion. The ultimate tensile strength is one of the essential tests within the industry to evaluate the material strength and also find the failure of materials [10,11,12,13]. There are several strength analyzing tests involved in industrial applications to maximize the strength of material which is to be used to fabricate components. In general, the tensile strength, compressive strength and shear strength tests are carried out by the way of using a universal testing machine [14,15,16]. Numerous testing procedures have to be followed to conduct the strength analyzing test effectively. From the stir casting process, the tensile strength of the hybrid composites are increased by the influence of process parameters, particularly stir casting time [17].

An increase in the stir casting time increases the tensile strength, as does increasing the percentage of reinforcement in the stir casting process. Hardness is one of the mechanical properties that offers a good material characteristic for the usage of industrial products [18,19,20,21]. Different types of hardness tests are available to find the hardness of the material such as Rockwell hardness, Brinell hardness test and Vickers hardness. Normally, the Brinell hardness test is used for heavy applications, with a 5–10 mm hard steel ball used as the indenter [22,23,24,25]. Accurate hardness measurement is also carried out by use of a microhardness test (Vickers hardness test), with a pyramid square diamond indenter used to measure the hardness of the material.

In all the moving parts, the wear has to occur promptly to reduce the surface structure of the parts and also to encourage the failure of the materials. Normally the wear test is conducted through wet and dry conditions based on the test procedure and the materials [26]. Dry sliding wear of Al8079–TiN/ZrO_2_ composites have high wear resistance compared to other combination composites. Wear rate can be dependent upon the various parameters, namely load, rpm, sliding distance and the adding of reinforcement. In industry, most machines suffer due to component wear. Use of loads and temperatures highly effect the wear rate of AMCs materials. In the Al8079–TiN/ZrO_2_ composites, lesser wear rates occurred with the applied load increases. In wear tests carried out on short fiber Saffil filled composites, it was observed that they had increased wear. When increasing the applied load, the wear and cracking resistance also increased. Similarly the wear rate was increased due to increase in the Al8079 temperature.

The corrosion property is one of the important roles in the marine environment and also in space applications. There are vast methods available for finding the corrosion resistance of materials within a short time period [27,28,29]. A few of the corrosion tests are: the electrochemical method, salt spray corrosion test, laboratory immersion corrosion test, etc. The corrosion test is conducted based on the pH values of the corrosion medium, such as an acidic or basic nature. Natural sea water is also a better corrosion medium for conducting corrosion tests at room temperatures and prominent temperature levels [30].

Sodium chloride is the best corrosion medium and provides a good result for the aluminum composite material. The novelty of this study is to fabricate aluminum composite materials such as AA8079 with reinforcement of titanium nitride (TiN) and zirconium oxide (ZrO_2_) by using the stirring casting route. Execution of the stir casting process with the influence of different parameters offered excellent casted hybrid composites. The stir casted samples were tested by namely the ultimate tensile strength test, wear test, microhardness test and salt spray test. All the test specimens were experimented with by influencing the different process parameters through the Taguchi method. This method provided the optimal parameters for obtaining good mechanical strength and corrosion resistance of the newly prepared hybrid composites [31].

The Al8079–TiN/ZrO_2_ composites had higher tension and wear properties due to the presence of both reinforced particles. These composites are widely used in aerospace and automotive industries for fabrication of components as well as structures.

## 2. Materials and Methods

Aluminum alloy 8079 possesses high strength as well as a highly machinable nature; these alloys are normally used in the automobile, aircraft and marine applications.

The chemical elements and their weight percentages present in the aluminum alloy 8079 material are represented in the Table 1. The mechanical strength values such as tensile strength, yield strength and fatigue strength of the base material AA8079 is illustrated in the Table 2.

The stir casting method is the leading process to enhance the properties of the composites with the use of any material in the casting furnace. The stir casting method is used for this research work to increase the mechanical strength as well as the corrosion resistance of the hybrid composites. This process was executed by observing different parameters: namely % of reinforcement, stirring speed and stirring time for the prepared exceptional composites.

## 3. Experimental Procedure

**Figure 1:**
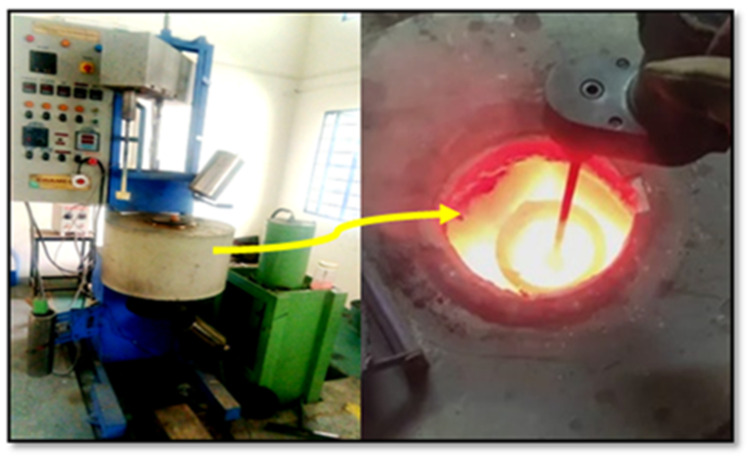
Stir casting process setup.

In this work, aluminum alloy (AA8079) with the reinforcement of titanium nitride and zirconium oxide was used for stir casting. Both the reinforced particles were utilized at different percentage levels (3%, 6% and 9%). The process parameters of this study were selected as % of reinforcement (3%, 6% and 9%), stirring time (20, 25 and 30 min) and stirring speed (500, 550 and 600 rpm). Figure 1 illustrates the entire stir casting setup of this investigation, the control panel having individual temperature adjustment regulator and speed control units [32,33,34]. The agitation motor can be controlled electrically for the use of stirring action. The furnace can be closed by the heat resistance cover to prevent the heat transfer to the atmosphere and provide safety for the work.

**Figure 2:**
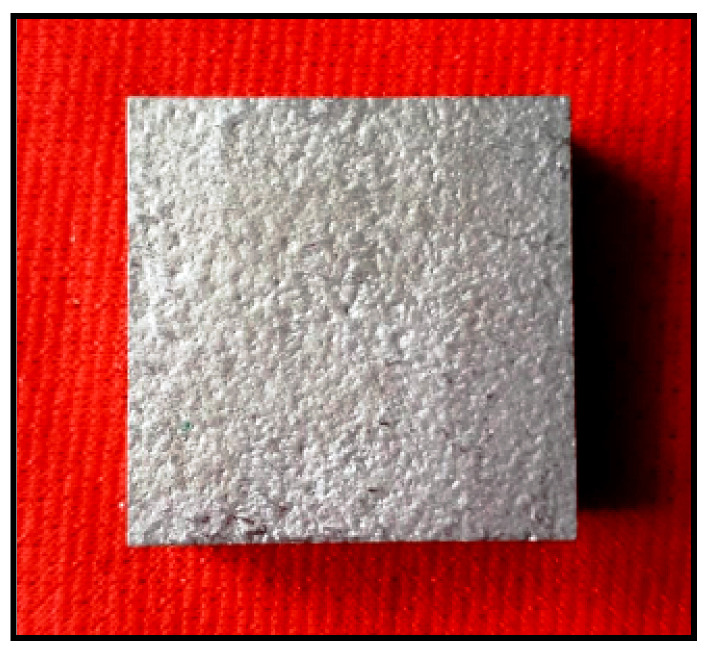
Stir casted AA8079 + TiN + ZrO_2_ samples.

Figure 2 illustrates the stir casted raw material of aluminum alloy and the reinforcement of titanium nitride and zirconium oxide. The samples are sliced out by use of a wire cut EDM machine with the required dimensions.

Table 3 presents the process parameters of the stir casting process, namely: percentage of reinforcement, stirring time and stirring speed. All three factors are chosen based on the conducting of mechanical tests such as the tensile test and wear test with different levels of process parameters.

Table 4 presents all the equipment and mentions the specifications of this experimental work. Different equipment is used, i.e., casting apparatus, universal testing machine, dry sliding wear test machine, salt spray testing machine and Vickers microhardness tester.

### 3.1. Ultimate Tensile Strength

**Figure 3:**
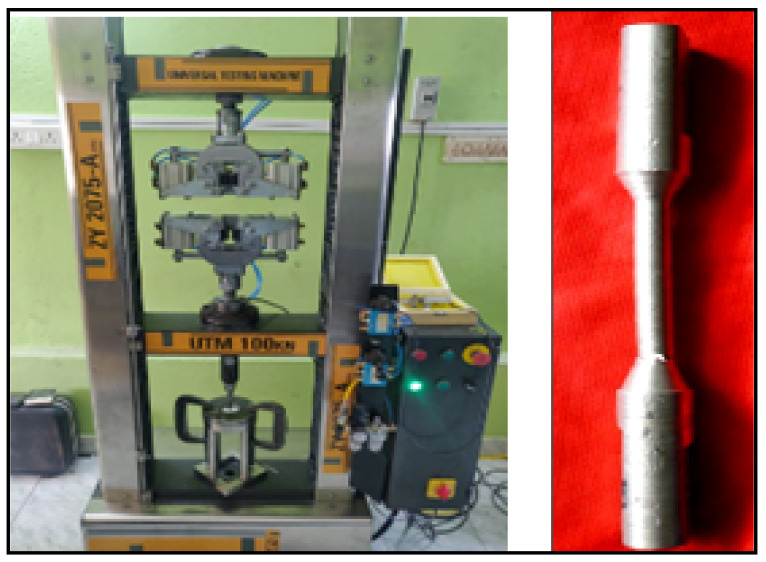
Schematic view of 100 kN capacity of UTM with tensile test specimen.

All the materials are checked for strength through some testing machine such as the tensile testing machine (universal testing machine). The UTM is generally used to measure the strength of the material such as tensile strength, compressive strength and shear strength, and all the tests are carried out in a single machine. Figure 3 shows the 100 kN capacity of the universal tensile testing machine (Model: ZY 2075–A International Equipments, Mumbai, India). The gripping jaws are operated pneumatically by using control levers and FRL units.

### 3.2. Wear Test

**Figure 4:**
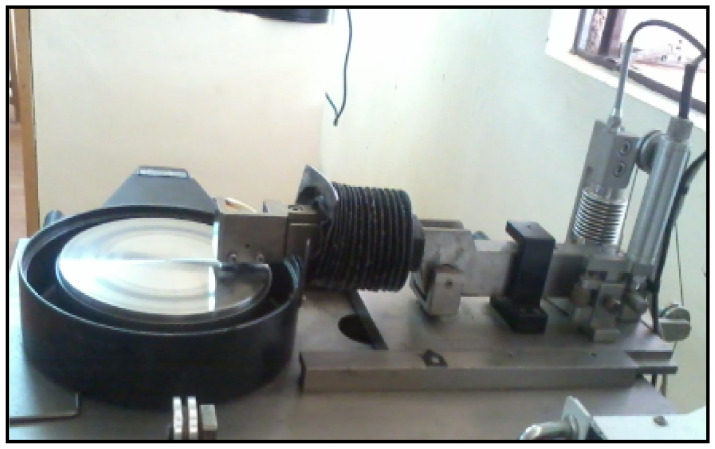
Schematic view of dry sliding wear test apparatus.

Hybrid composite specimens wear was analyzed through a dry sliding wear test apparatus, as shown in Figure 4. The specimens were prepared from the raw stir casted hybrid composites. The pins are prepared as per the ASTM G99–05 standard, the dimensions of the specimens are 30 mm height and 12 mm diameter. The rotating disc was made on EN30 steel with hardness value of 60 HRC, the standard test procedure was implemented for this study. The parameters were selected as sliding velocity of 3 m/s, applied load of 30 N and sliding distance of 1500 m. The pins are polished and cleaned well before conducting of wear test. The average value of wear readings are noted from the DUCOM Instruments, Bengaluru, India dry sliding wear test machine for each specimen. The weights of the pins are measured before and after conducting of the wear test.

### 3.3. Corrosion Test

The corrosion test process factors and their levels are presented in Table 5; the parameters are taken as reinforcement percentage, pH value of the corrosion medium and the hang time of the specimens.

**Figure 5:**
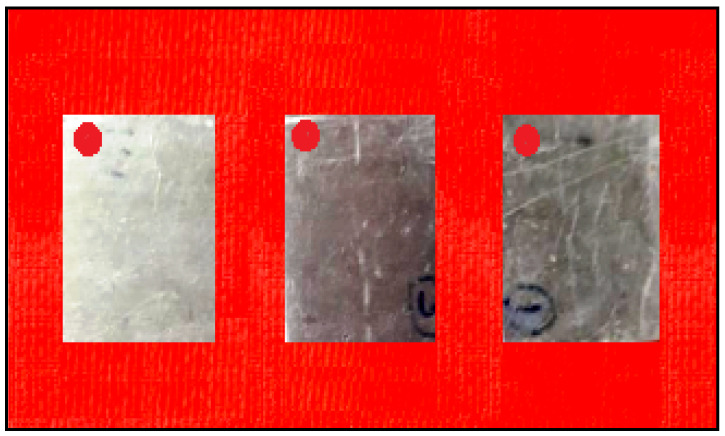
Photographic view of corrosion specimens.

The nine samples are prepared by the dimensions 20 × 15 × 8 mm from the sintered compaction samples and the specimens are cleaned and allowed to dry as shown in Figure 5. Initially, all samples were weighed and their initial weight was noted for estimating the mass loss of the specimens during the salt spray test. All the specimens were fitted by means of being hung in the chamber, with the help of small holes made in the samples. A NaCl solution of 3% was circulated at the maintained temperature of 35 °C for spraying on the specimens with different pH value concentrations [35,36,37]. The time period of the experiment was maintained for 24, 48 and 72 h, then after the time period the specimens were taken out, dried, weighed and finally calculated for the corrosion rate of the specimens [38].

### 3.4. Microhardness Test

**Figure 6:**
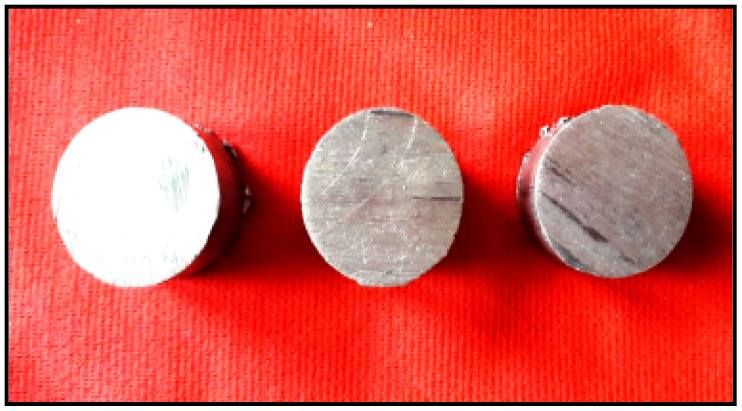
Photographic view of microhardness specimens.

Microhardness of the hybrid composites was conducted by using the Vickers hardness tester. The specimens are prepared as per the ASTM standard (American Society for Testing and Materials.) at 15 mm height and 25 mm diameter using the CNC machine Machine Tools, Chennai, India. The Figure 6 illustrates the microhardness test specimens. Before conducting the microhardness tests, the specimens were cleaned and polished well [39,40]. All the specimens from the salt spray test were utilized to conduct the microhardness tests. The optimization parameters were percentage of reinforcement, pH value and hang time in hours.

All the response values of the test can be checked with the S/N ratio, such as the signal-to-noise ratio. It is demonstrated as the ratio of the power of a signal (meaningful information) and the power of background noise (unwanted signal). The significance of precisely calculating the signal to noise ratio is necessary for the decisive goal of efficient and accurate designs.

### 3.5. SEM Analysis

In this investigation the ZEISS brand (Model: Sigma, St. Louis, MO, USA) scanning electron microscope (SEM, OXFORD Instruments India Pvt. Ltd., Mumbai, India) was used to examine the wear and corrosion effects of the composite specimens. High magnification can be obtained from this instrument such as 15×~300,000× and the acceleration voltage is 1~30 kV. High resolution is possible for use of this instrument, such as 3.0 nm (at 30 KV, SEM Image). All the samples were tested effectively; before conducting the SEM analysis the specimens were cleaned well, sliced out at the correct size and polished to a high level. The polishing has to be performed via use of a rotating polishing disc.

## 4. Result and Discussion

### 4.1. Ultimate Tensile Strength

Table 6 presents the experimental summary of ultimate tensile strength; three factors contributed their influence and produced a different range of ultimate tensile strengths. The maximum ultimate tensile strength was obtained as 205.52 MPa from the sixth experimental runs. It is achieved by the influence of 6% of reinforcement, 30 min of stirring time and 500 rpm of stirring speed. The minimum ultimate tensile strength was observed as 185.67 MPa. The highest S/N ratio and lowest S/N ratio were presented as 46.2571 and 45.3748, respectively.

**Figure 7:**
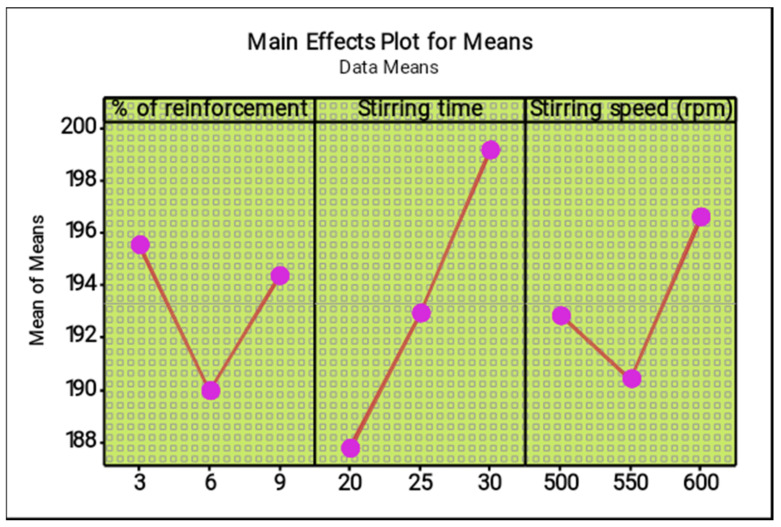
Main effect plot for means (ultimate tensile strength).

**Figure 8:**
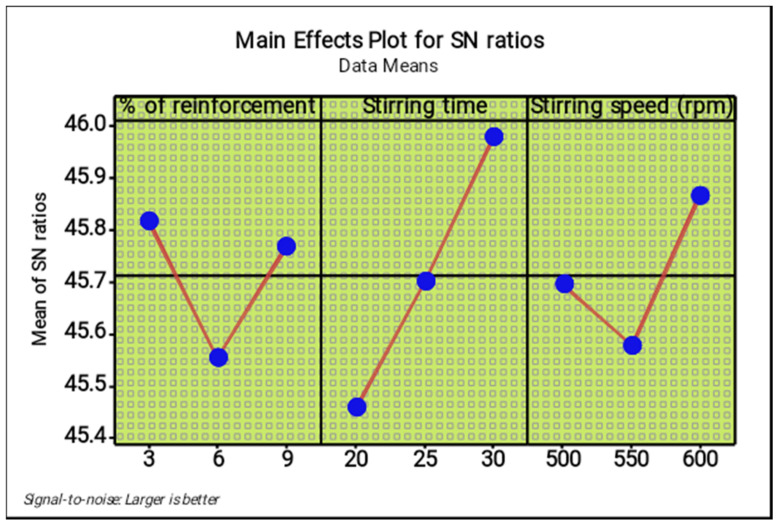
Main effect plot for S/N ratios (ultimate tensile strength).

Table 7 and Table 8 present the response tables for the means and signal to noise ratios of the ultimate tensile strength respectively. From the tables, the stirring time was identified as the most influence factor, followed by stirring speed and percentage of reinforcement. Optimal parameters of the test were found as 3% of reinforcement, 30 min of stirring time and 600 rpm of stirring speed. Figure 7 and Figure 8 illustrate the main effect plots for the mean and S/N ratio of the ultimate tensile strength, respectively. From the three parameters, 3% of reinforcement offered the maximum ultimate tensile strength, and 3% to 6% of reinforcement reduced the ultimate tensile strength. Further increasing from 6% to 9% slightly increased the ultimate tensile strength.

The increase in stirring time proved that the maximum ultimate tensile strength can be increased, such as by increasing the stirring time from 20 to 30 min. The maximum stirring time of 30 min offered excellent ultimate tensile strength. The lowest stirring speed decreases the tensile strength, while an increase in stirring speed from 500 rpm to 600 rpm increased tensile strength moderately. The 600 rpm speed provided the maximum tensile strength.

**Figure 9:**
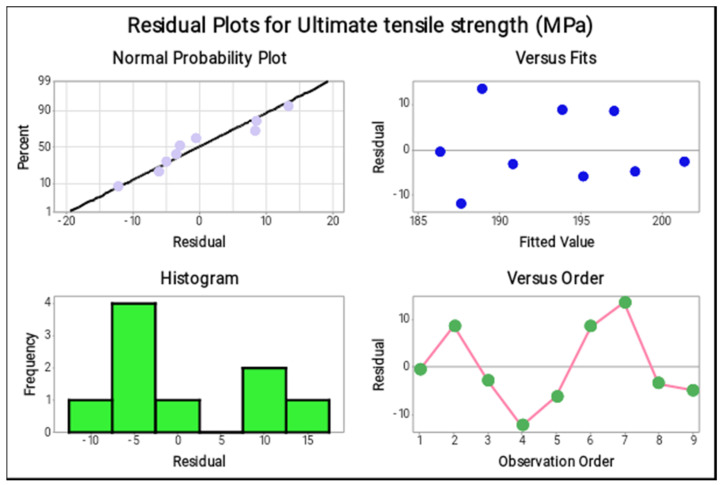
Residual plots (Ultimate tensile strength).

The four in one plots (Figure 9), such as the residual plot, indicated which parameters are to be highly involved to produce the maximum tensile strength. In a normal probability plot, most of the points are lying on the mean line and few of them close to the mean line; thereby, it is observed that the chosen parameters are excellent and that the ultimate tensile strength was accurate. In the versus fits plot, the nine points are distributed in a normal condition and the distribution values are lying within the limit. The histogram plots versus the order plots provided the accurate results of the ultimate tensile strengths. The points were distributed in a rectangle form and the zigzag motions proved that the selection of parameters highly reflected the response values.

Table 9 presents the analysis of variance for the ultimate tensile strength; this statistical analysis provides the contribution of each parameter in the ultimate tensile strength test. The highest contribution was observed as 24.94% by stirring time, followed by stirring speed (2.71%) and percentage of reinforcement (0.26%). The *p*-values were obtained in normal range, it also informed the selected parameters are excellent.

### 4.2. Wear Test

Table 10 illustrates the experimental summary of the wear test; all three process parameters are bequeathing their influence and formulate a various range of wear values. In the fifth experimental run, the minimum wear value was found to be 119.53 µm. This minimum wear value was observed by the influence of 6% of reinforcement, 25 min of stirring time and 600 rpm of stirring speed. Contrarily, the maximum wear was recorded as 147.26 µm. Similarly, the highest S/N ratio and lowest S/N ratio were registered as −43.3617 and −41.5495, respectively. All the nine experimental runs were effectively conducted so that each parameter was shown to contribute its individual influence.

Table 11 and Table 12 presented the response table for means and signal to noise ratios of the wear respectively. From these tables, the reinforcement percentage was observed as the prime factor of the wear test, followed by the stirring speed and finally the stirring time. In the wear test the optimum parameters were concluded to be: 9% of reinforcement, 25 min of stirring time and 550 rpm of stirring speed.

**Figure 10:**
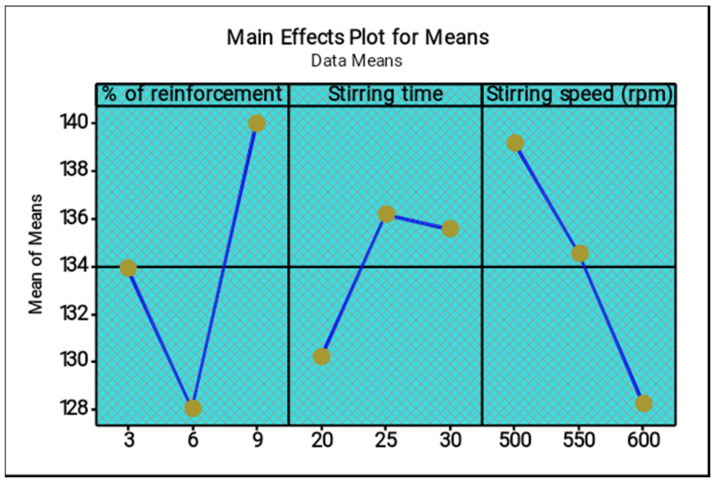
Main effect plot for means (wear).

**Figure 11:**
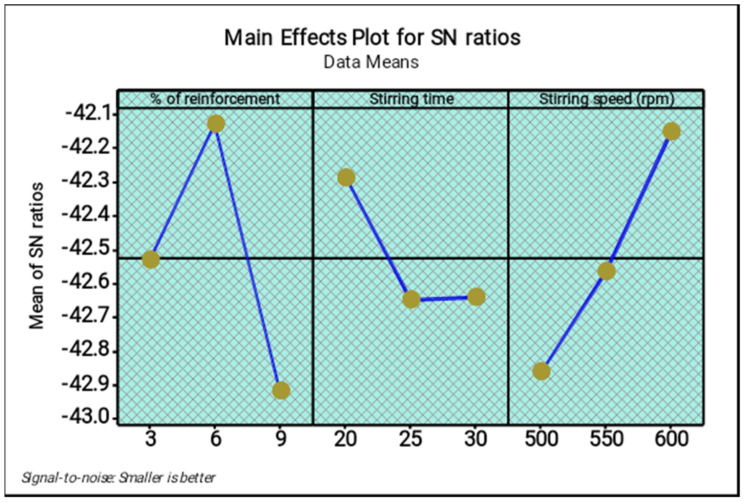
Main effect plot for S/N ratios (wear).

Figure 10 and Figure 11 show the main effect plots for the mean and S/N ratio of the wear test, respectively. Among the three parameters, 6% of reinforcement offered the minimum wear values, whereas 6% to 9% of reinforcement increases the wear values such that the wear resistance of the composites were reduced. Three percent of reinforcement produced a moderate wear condition. The minimum range of the stirring time (20 min) offered the high wear resistance compared to other stirring times. Increase in the stirring time demonstrated that the maximum wear occurred. The moderate stirring time of 25 min produced poor wear resistance. The highest stirring speed increased the wear resistance; increasing the stirring speed from 500 to 600 rpm led to an increase in the wear resistance. The 600 rpm speed provided the maximum wear resistance of the composites. The minimum stirring speed offered the maximum wear.

**Figure 12:**
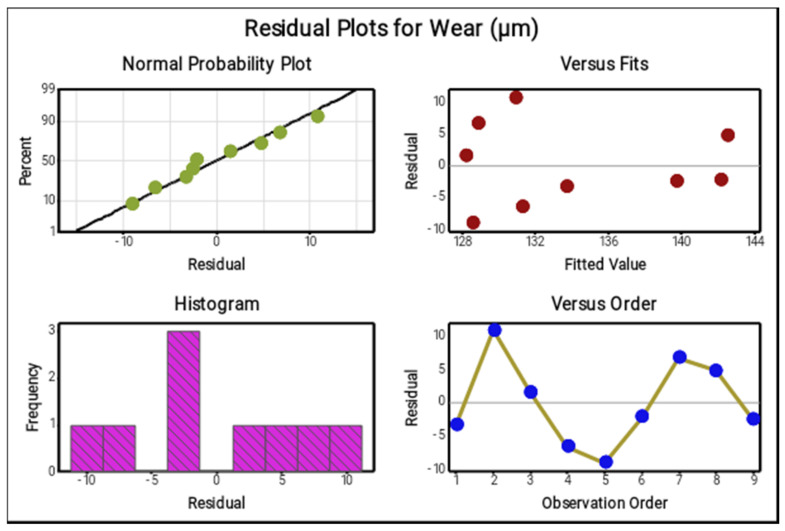
Residual plots for wear.

The four in one plot in Figure 12 specified the parameters that had extreme effect in forming the minimum wear values of the composites. In the regular probability plot the majority of the points are lying on the mean line, few of them are incredibly near to the mean line, therefore it can be seen that the preferred parameters are outstanding and exactly obtained the minimum wear values. In the versus fits plot, the nine points are scattered in a regular condition and the scattered values are lying inside the limit. The histogram and versus order plots offer the precise results of the minimum wear values. From these plots, the points are dispersed in a rectangle appearance and zigzag motion, confirming that the preferred parameters exceedingly replicate the response values.

Table 13 illustrates the analysis of variance for the wear test; this numerical analysis provides the involvement of each parameter in the minimum wear test. The maximum contribution was observed as: 29.14% stirring speed, followed by percentage of reinforcement (9.10%) and stirring time (7.11%). Probability values such as the *p*-values were gained in the minimum range, and it was also observed that the selected parameters were exceptional.

**Figure 13:**
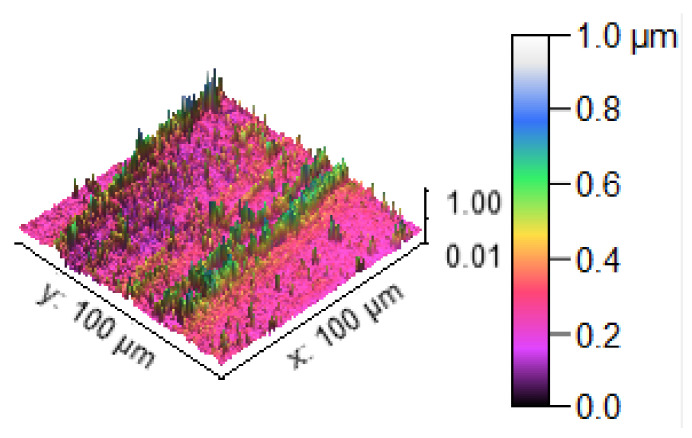
Three dimensional (3D) profilometric image (S-1).

**Figure 14:**
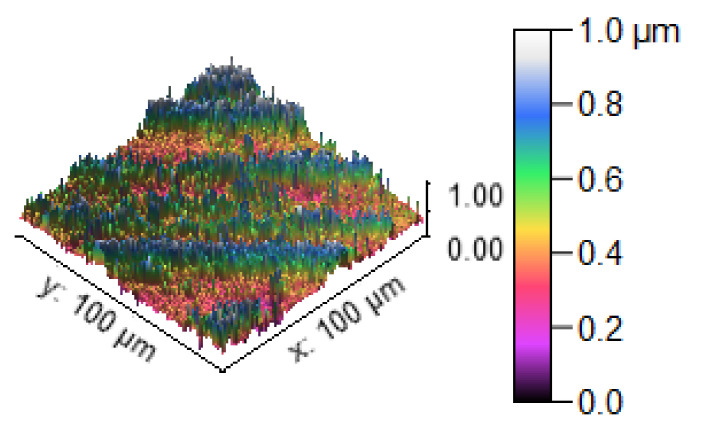
Three dimensional (3D) profilometric image (S-2).

**Figure 15:**
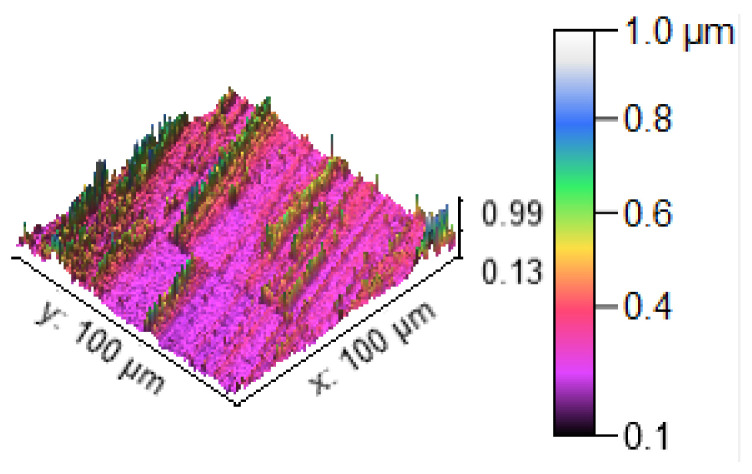
Three dimensional (3D) profilometric image (S-3).

**Figure 16:**
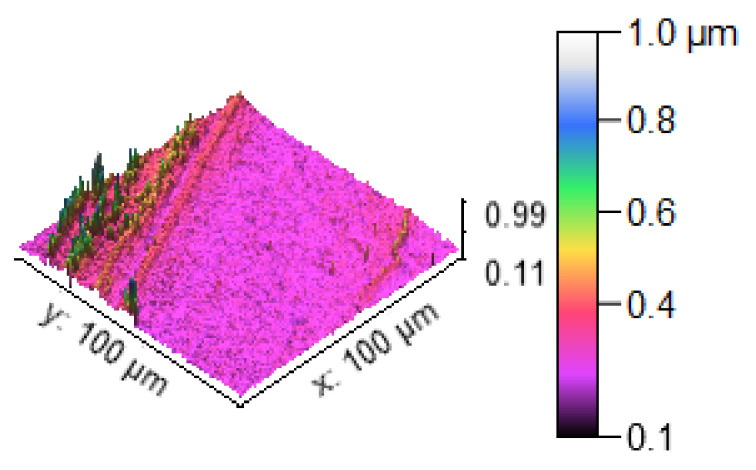
Three dimensional (3D) profilometric image (S-4).

**Figure 17:**
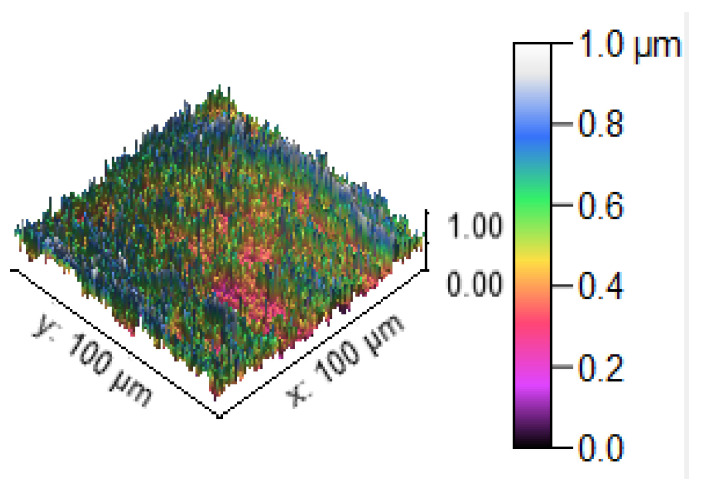
Three dimensional (3D) profilometric image (S-5).

**Figure 18:**
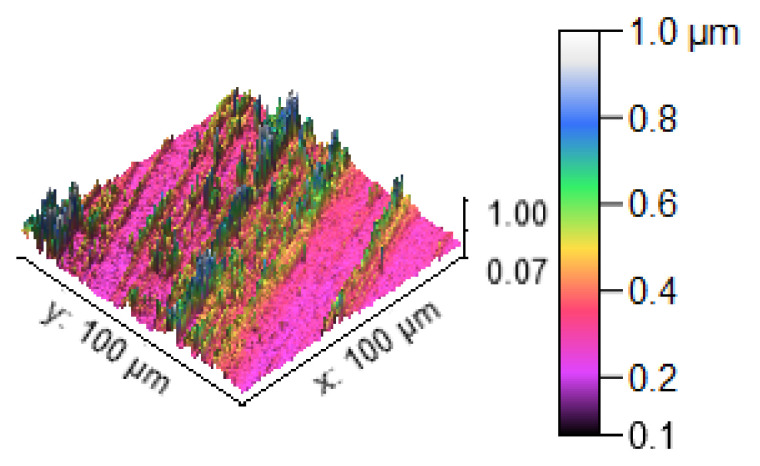
Three dimensional (3D) profilometric image (S-6).

Figure 13 illustrates the 3D profilometric image of the first specimen; the wear has highly occurred in the surface of the specimen; it can be denoted in the pink colour with below 0.4 microns level. Figure 14 shows the minimum wear, as proven by the maximum surfaces resistance to wear by the way of the greenish blue color. The wear can be maintained at the level of 0.4 to 0.8 microns. Figure 15 and Figure 16 illustrate the highly affected wear surface of the specimens, where the maximum wear was obtained in the surfaces via the influence of minimum stirring speed and minimum stirring time. Figure 17 presents the high quality of wear resistance surface of the specimen; this shows that a small amount of wear can be obtained. Figure 18 shows the high wear rate that occurred in a partial area of the specimen, as the middle portion of the specimen surface offered resistance to wear.

**Figure 19:**
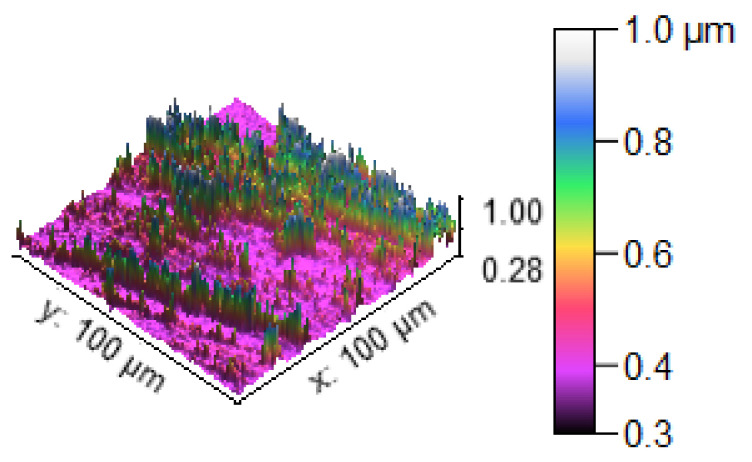
Three dimensional (3D) profilometric image (S-7).

**Figure 20:**
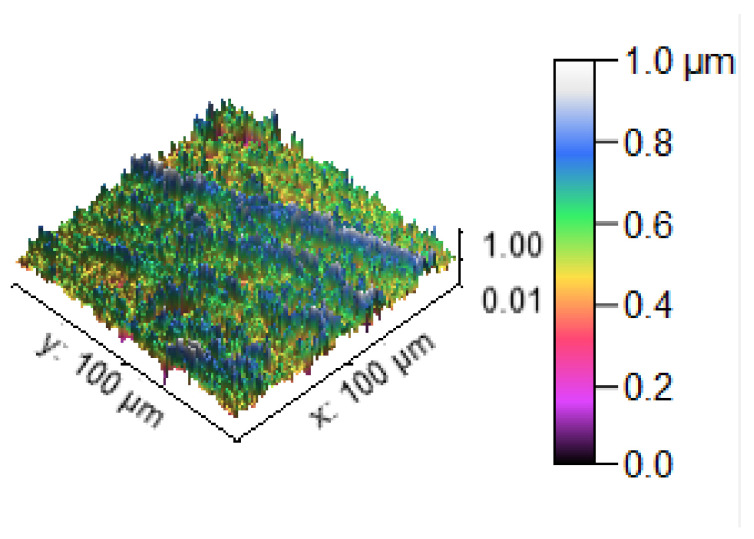
Three dimensional (3D) profilometric image (S-8).

**Figure 21:**
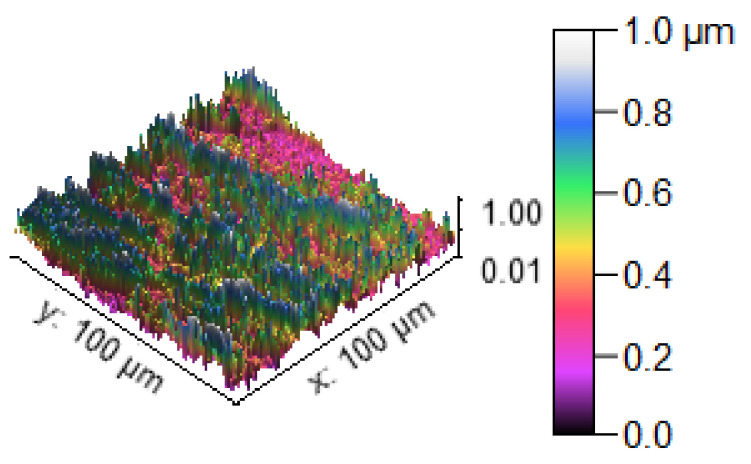
Three dimensional (3D) profilometric image (S-9).

Figure 19 illustrates the extreme wear occurrence on the surface of the specimen due to the varying stirring speed. Figure 20 presents the microns level of wear taking place on the specimen surface, showing that the specimen has high resistance to wear even at low stirring time. Figure 21 represents that moderate wear can take place on the specimen, with only a small surface area affected by the wear and with the remaining area unaffected.

### 4.3. Salt Spray Corrosion Test

The corrosion test input factors (percentage of reinforcement, pH value and Hang time), output measurement of corrosion rate and the signal to noise ratio values (hours) are presented in Table 14.

The hanging time is the first influencing factor of this study, astabulated in Table 15 and Table 16. The pH value is the second rank of this study and the percentage of reinforcement is the third order of the research. The optimal values found from this analysis were: the first level of reinforcement—percentage (6%), second level—pH value (3) and third level—hang time (24 h).

**Figure 22:**
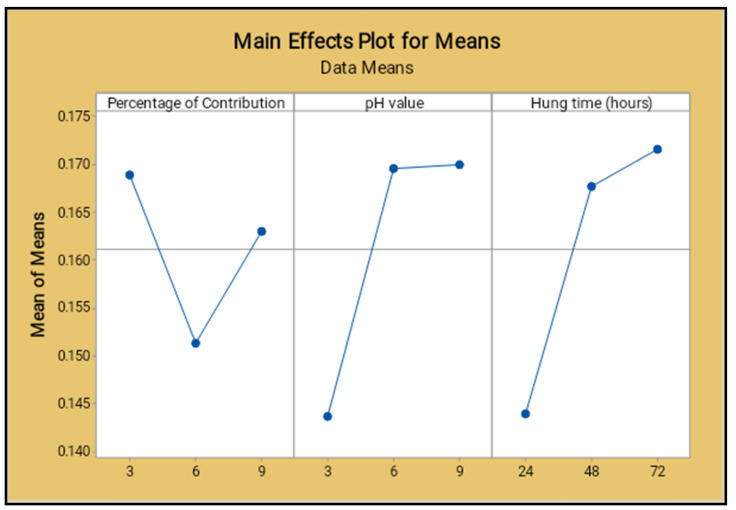
Main effect plot for means (salt spray test).

**Figure 23:**
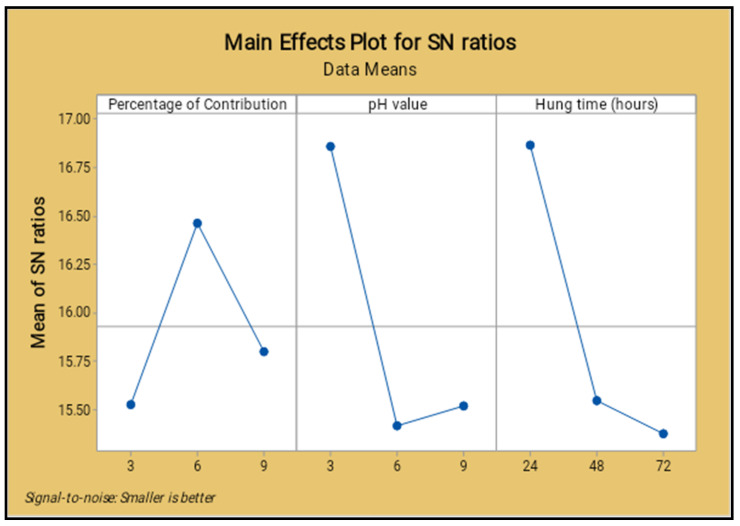
Main effect plot for signal to noise ratio (salt spray test).

The lowest percentage level of reinforcement produced the minimum corrosion rate, the middle level of the pH value reduced the corrosion rate, increased the operation time (hang time) also decreased the corrosion rate, as shown in Figure 22 and Figure 23. The 6% of reinforcement offered the minimum corrosion, while with further increasing 6% to 9% the corrosion rate also increased. The lowest pH value produced the minimum corrosion, i.e., the 3 pH value offered minimum corrosion, while with further increase of the 3 pH value to 9 pH the corrosion rate also increased. The maximum corrosion rate was found to be using the 9 pH value; similarly, the minimum hang time period offered the least corrosion level. By continually increasing the hang time period from 24 h to 72 h, the maximum corrosion occurred at the 72 h time period.

**Figure 24:**
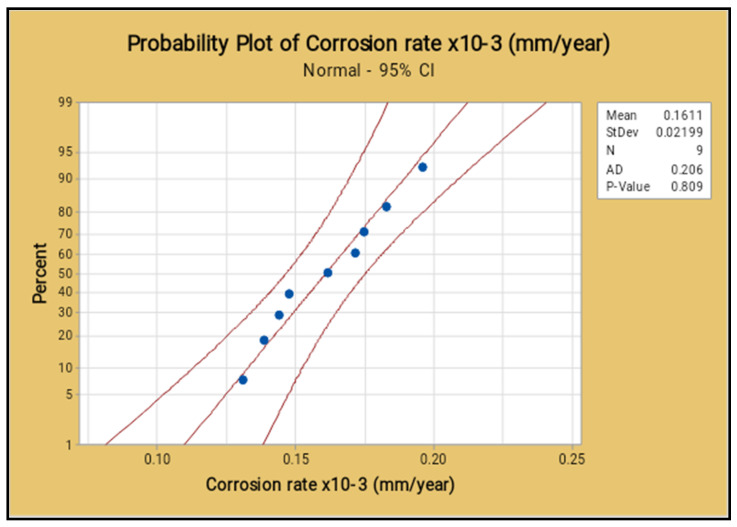
Probability plot for corrosion rate.

The normal probability of the corrosion rate analysis for all selected factors are placed closer to the mean line and few of them deviated, as shown in the Figure 24. It proves that all nine process parameters of this study are exceptional ones.

Table 17 presents the factors contribution percentages clearly, and the hang time contributed a higher percentage level (57.96%) compared to the other factors. The parameter of the pH value contributed 29.68%, the reinforcement percentage contribution was 12.36%, and the higher value of Fisher ratio (3.53) obtained for the hang time is also illustrated in the table. The probability values of all parameters were in minimum in range.

**Figure 25:**
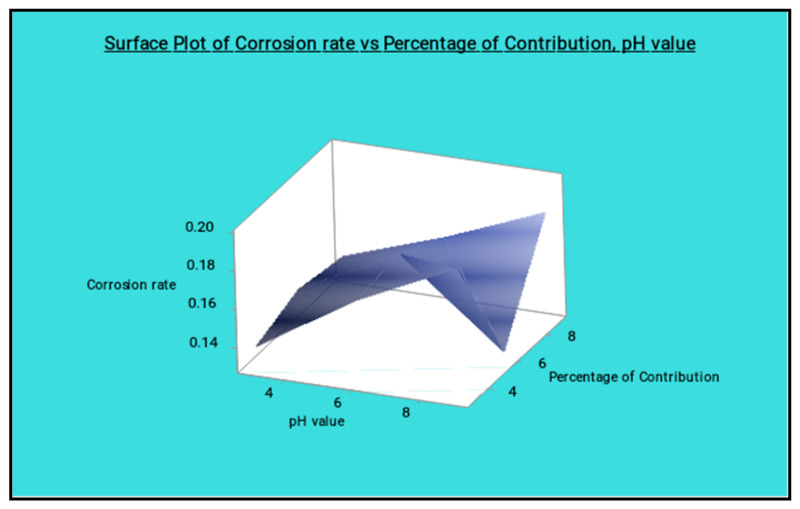
Surface plot of corrosion rate vs. percentage of reinforcement and pH value.

**Figure 26:**
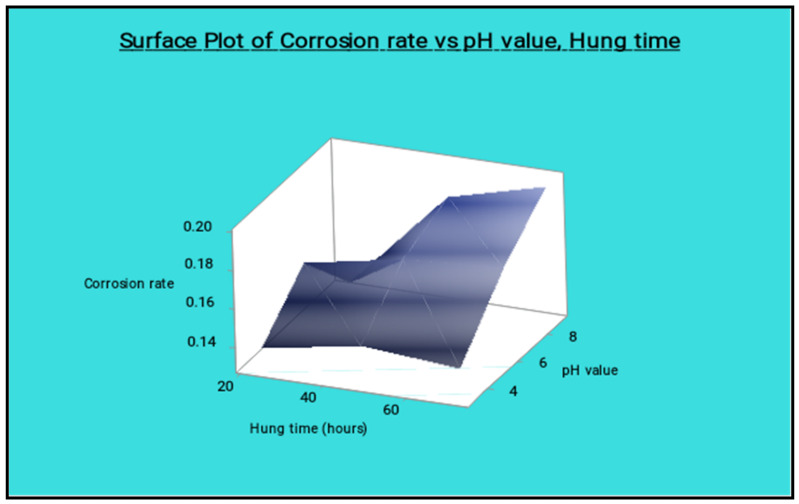
Surface plot of corrosion rate vs. pH value and hang time.

Figure 25 shows the surface plot of the corrosion rate vs. percentage of reinforcement and pH value visibly; from this plot the low percentage of reinforcement and the minimum pH value produces the minimum corrosion rate. Figure 26 shows the surface plot of corrosion rate vs. pH value and hang time clearly; in this plot it is indicated that the maximum hang time and minimum pH value offered the minimum corrosion rate. The minimum pH value and the minimum hang time also give less corrosion rate.

**Figure 27:**
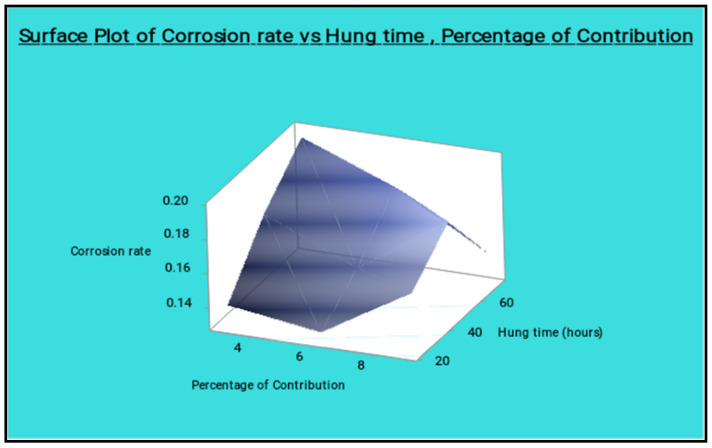
Surface plot of corrosion rate vs. hang time, percentage of reinforcement.

The minimum percentage of reinforcement and the slightly increasing hang time gives the lowest corrosion rate, as shown in the surface plot Figure 27. The moderate level of the reinforcement and the lowest level of the hang time present the lowest corrosion rate.

#### Microstructure Analysis

**Figure 28:**
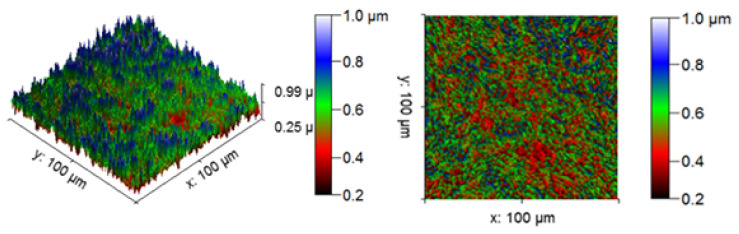
Corrosion test specimen (minimum corrosion).

**Figure 29:**
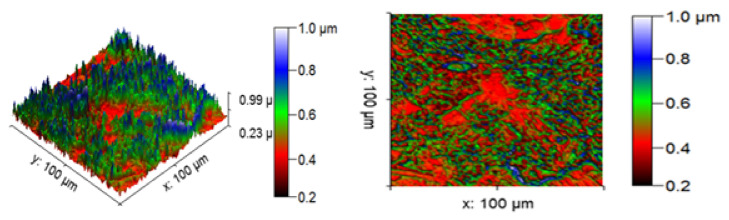
Corrosion test specimen (moderate corrosion).

**Figure 30:**
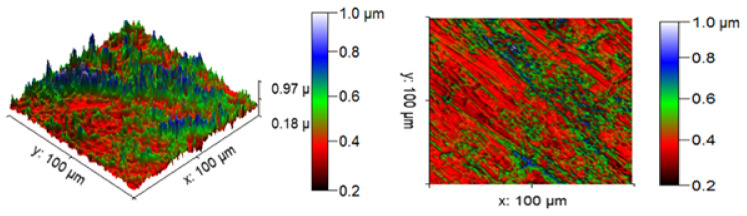
Corrosion test specimen (aximum corrosion).

The SEM image of the corrosion test specimens were changed into the 2D and 3D profilometry images, as shown in Figure 28, Figure 29 and Figure 30. Figure 28 shows the minimum corrosion rate that occurred; the blue and green colors indicate the minimum corrosion takes place on the surface of the specimen. The colour bar indicates that the 0.6 to 1.0 microns corrosion resistant level can be maintained. The small amount of corrosion that takes place in the specimen is illustrated in the 2D view in the form of the color red. Figure 29 shows that moderate corrosion can take place on the specimen due to the increasing hang time in the corrosion test. The 2D image evidently illustrates the fairly corroded area of the specimen with the indication of the color red. Most of the area can be corroded severely, as reflected in Figure 30, and a high PH value of the corrosion medium can directly produce a high corrosion. The maximum area can be seen at the 0.2 to 0.4 levels of corrosion, confirming the corrosion influence in the experimental work. The 2D image reveals the high corrosion rate in the specimen by the way of showing maximum of the color red.

### 4.4. Microhardness Test

Table 18 presents the experimental summary of the microhardness test, using the three process parameters influencing the different ranges of microhardness values. From the analysis, the maximum microhardness value was obtained as 155 HV from the fifth experimental runs. It is accomplished by the influence of 6% of reinforcement, pH value of 6 and 72 h of hang time. The minimum microhardness value was observed as 134 HV. The highest S/N ratio and lowest S/N ratio were presented as 43.8066 and 42.5421, respectively.

Table 19 and Table 20 present the response table for means and signal to noise ratios of the corrosion test respectively. From the tables, the percentage of reinforcement was identified as the most significant influence parameter, followed by the pH value parameter and hang time. The optimal parameters of the microhardness test were registered as: 6% of reinforcement, pH value of 6 and 48 h of hang time. 

**Figure 31:**
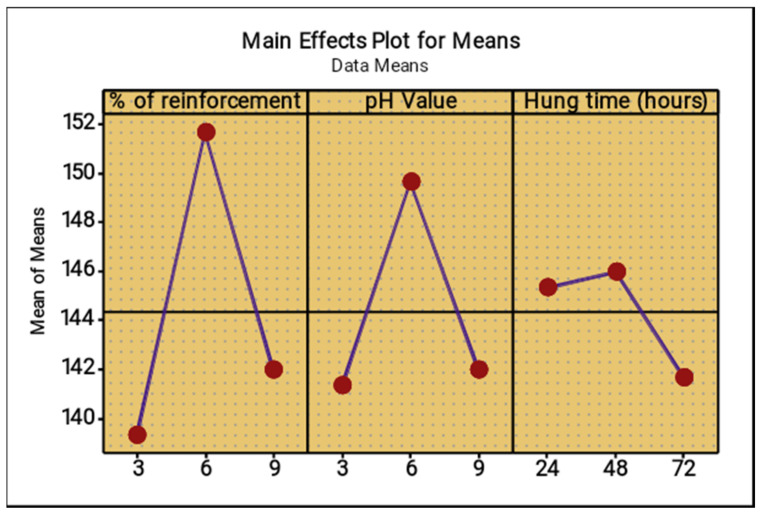
Main effect plot for means (microhardness).

**Figure 32:**
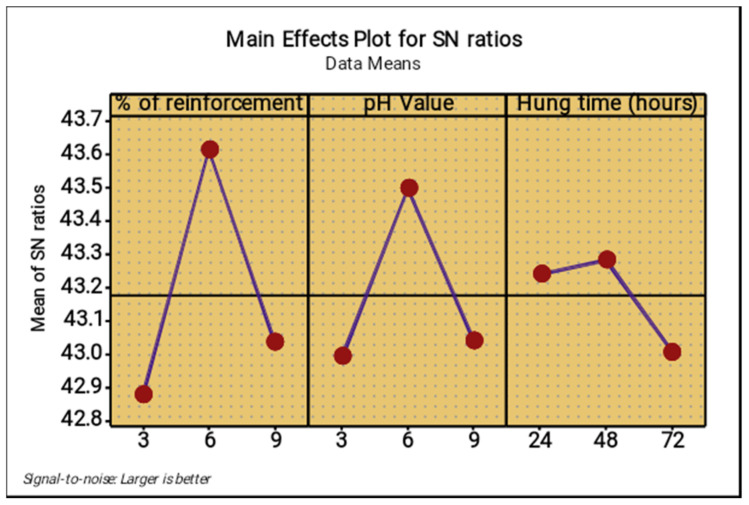
Main effect plot for S/N ratios (microhardness).

Figure 31 and Figure 32 present the main effect plots for mean and S/N ratio of the microhardness test, respectively. From three parameters, 6% of reinforcement offered the maximum microhardness value, whereas 6% to 9% of reinforcement reduced the microhardness value. Minimum microhardness was observed at 3% of reinforcement. A moderate pH value (6) offered excellent microhardness values, whereas further increase in the pH value from 6 to 9 reduced the microhardness value. A low pH value (3) offered a minimum hardness value. A hang time of 48 h offered an exceptional microhardness value. With an increase in hang time from 48 h to 72 h, the microhardness value decreased. The hang time of 72 h offered the lowest microhardness value.

**Figure 33:**
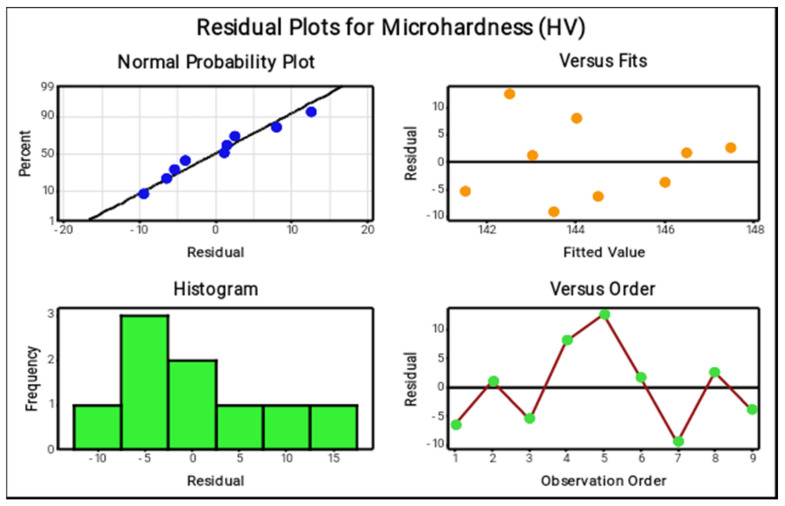
Residual plots for microhardness.

The residual plots indicated which parameters were most effective at forming the maximum microhardness. In the normal probability plot, nearly every one of the points e lie on the mean line and only a small number of points are extremely near to the mean line; thus, the elected parameters are extraordinary and accurately obtain the microhardness. In the versus fits plot, the nine points are scattered in a standard condition and the distributed values are lying inside the limit. In Figure 33, The histogram and versus order plots present the precise result of the microhardness; the points are spread in a rectangle figure and zig-zag motion, confirming that the choice of parameters reflected the response values well.

Table 21 illustrates the analysis of variance for the microhardness test; this statistical analysis gives the contribution of each parameter in the microhardness test. The maximum contribution was observed as 4.585% by hang time, followed by percentage of reinforcement (2.42%) and pH value (0.15%). The *p*-values were obtained in the normal collection; this also informs that the chosen parameters are remarkable ones [41,42,43,44,45,46,47,48,49].

### 4.5. Scanning Electron Microscopy Analysis

**Figure 34:**
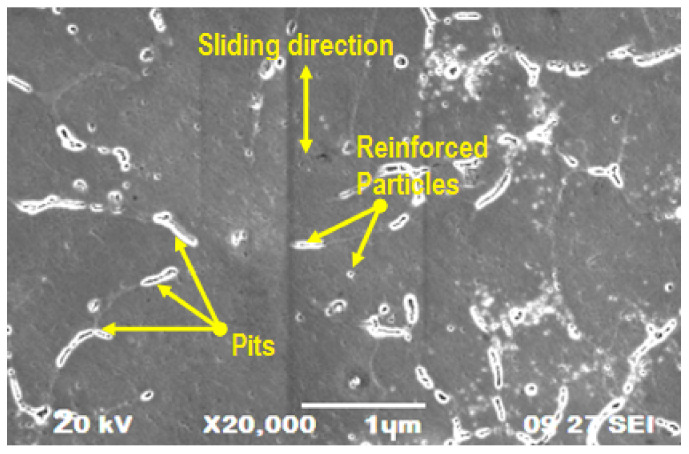
SEM image of worn out wear specimen.

**Figure 35:**
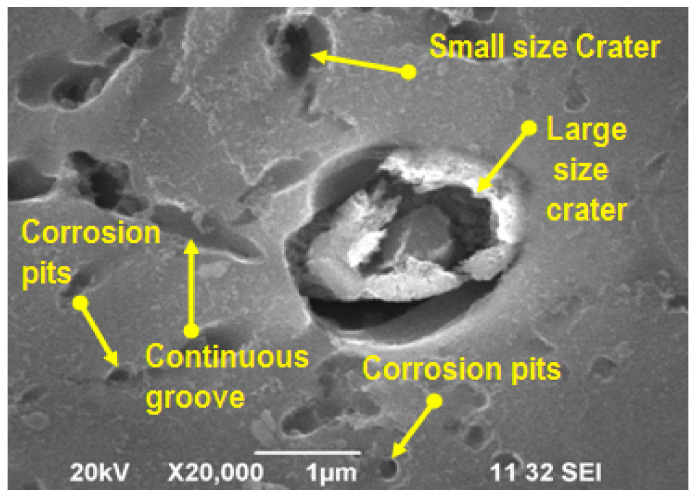
SEM image of corrosion specimen.

Scanning electron microscopy was used to analyze the topographic characterization of the composite materials in an effective manner. Figure 34 and Figure 35 were illustrate the SEM image of wear and corrosion in the specimen. Figure 34 represents the higher wear rate observed in the specimen, where high applying load and speed caused the large number of pits due to heat generation between specimen and disc. Nonuniform blending of the reinforced particles are clearly shown in the SEM image. Figure 35 presents the SEM image of the highly corroded specimen. This image illustrated the corrosion pits, various sizes of craters and continuous grooves. These defects were formed by the influence of more hanging time; similarly, the non-homogeneous mixture of the reinforced particles produced higher craters in particular areas of the specimen.

## 5. Conclusions

In this work, aluminum alloy with the addition of titanium nitride and zirconium oxide hybrid composites were prepared through the stir casting methodology, and the composites were tested by the tensile test, wear test, corrosion test and microhardness test. All the test parameters were optimized and the result of this work was summarized as follows:

- From the ultimate tensile strength the maximum ultimate tensile strength was observed as 205.52 MPa from the sixth experimental runs. It was achieved by the influence of 6% of reinforcement, 30 min of stirring time and 500 rpm of stirring speed. Reinforcement led to increase the ultimate tensile strength due to the higher strength of the reinforced particles used in the composite preparation. Uniform mixing also provided an increase in the ultimate tensile strength of the specimen. The optimal parameters of the tensile test were found as 3% of reinforcement, 30 min of stirring time and 600 rpm of stirring speed.
- In the wear test, the minimum wear value was found to be 119.53 µm, as observed in the fifth experimental run. This minimum wear value was observed by the influence of 6% of reinforcement, 25 min of stirring time and 600 rpm of stirring speed. By contrast, the maximum wear was recorded at 147.26 µm. High stirring speed and stirring time was influenced to form a homogeneous mixture of the composites, causing the minimum wear of the specimen. The higher stirring speed offered a uniform and extreme blending of the reinforced particles with the base materials. In the wear test, the optimum parameters were concluded as 9% of reinforcement, 25 min of stirring time and 550 rpm of stirring speed.
- From the entire work, the hang time was the most significant factor of the corrosion analysis, followed by the pH value and reinforcement percentage. The most favorable values of this experiment were obtained as: 6% of reinforcement, pH value of 3 and hang time of 24 hrs. Increases in hang time and pH values increased the corrosion rate due to the high chemical action affecting the structure of the materials. The hang time was maximally contributed as 57.96%, the next level of contribution was 29.68% from the pH value, and finally the reinforcement of titanium nitride particles contributed 12.36%.
- From the analysis, the maximum microhardness value was obtained as 155 HV from the fifth experimental runs. It was accomplished by the influence of 6% of reinforcement, pH value of 6 and 72 h of hang time. The minimum microhardness value was observed as 134 HV. The optimal parameters of the microhardness test were registered as 6% of reinforcement, pH value of 6 and 48 h of hang time. A moderate range of pH value and reinforcement percentage increases the microhardness.

## Figures and Tables

**Table 1:** Chemical constituent of AA 8079.

Material	Composition in %
Copper	0.05
Iron	1.3
Zinc	0.10
Silicon	0.30
Other	0.15
Aluminum (Al)	Remaining

**Table 2:** Mechanical strength of AA 8079.

Properties	Metric
Tensile strength	150 MPa
Yield strength	120 MPa
Fatigue strength	56 MPa
Elongation	2.2%

**Table 3:** Process parameters and their levels of stir casting process.

S. No	Parameters	Level 1	Level 2	Level 3
1.	% of reinforcement	3	6	9
2.	Stirring time	20	25	30
3.	Stirring speed	500	550	600

**Table 4:** Equipment used for testing and specifications.

Equipment Used	Specification
Stir casting apparatus	Bottom pouring type Maximum operating temperature 1000 °C Stirring speed:100 to 1500 rpm
Universal Testing Machine (UTM)	ZY 2075—A series, 100 kN capacity
Dry sliding wear test machine	DUCOM Model TR-20-M-106, Disc material: EN-8 medium carbon steel
Salt spray testing machine	Brand: Weiss, Material: Stainless Steel, Frequency level: 70 Hz, Voltage: 230 V Power: Electric, Size: 500 × 300 × 500
Vickers microhardness tester	Model: V-5 series, Range: 1 HV-2967 HV, Eyepiece: 10×, Dimension: 500 × 250 × 650 mm

**Table 5:** Process variables and their levels (corrosion test).

S. No	Parameters	Level 1	Level 2	Level 3
1.	Percentage of reinforcement	3	6	9
2.	pH value	3	6	9
3.	Hang time (hours)	24	48	72

**Table 6:** Experimental summary of ultimate tensile strength.

S. No	% of Reinforcement	Stirring Time	Stirring Speed (rpm)	Ultimate Tensile Strength (MPa)	S/N Ratio
1	3	20	500	185.67	45.3748
2	3	25	550	202.42	46.1251
3	3	30	600	198.47	45.9539
4	6	20	550	175.39	44.8801
5	6	25	600	188.96	45.5274
6	6	30	500	205.52	46.2571
7	9	20	600	202.31	46.1203
8	9	25	500	187.34	45.1203
9	9	30	550	193.41	45.4526

**Table 7:** Response table for means (ultimate tensile strength).

Level	% of Reinforcement	Stirring Time	Stirring Speed (rpm)
1	195.5	187.8	192.8
2	190.0	192.9	190.4
3	194.4	199.1	196.6
Delta	5.6	11.3	6.2
Rank	3	1	2

**Table 8:** Response table for signal to noise ratios (ultimate tensile strength).

Level	% of Reinforcement	Stirring Time	Stirring Speed (rpm)
1	45.82	45.46	45.69
2	45.55	45.70	45.58
3	45.77	45.98	45.87
Delta	0.26	0.52	0.29
Rank	3	1	2

**Table 9:** Analysis of variance for ultimate tensile strength.

Source	DF	Seq SS	Contribution	Adj SS	Adj MS	F-Value	*p*-Value
Regression	3	215.993	27.91%	215.993	71.998	0.65	0.619
% of reinforcement	1	2.042	0.26%	2.042	2.042	0.02	0.898
Stirring time	1	193.007	24.94%	193.007	193.007	1.73	0.245
Stirring speed (rpm)	1	20.944	2.71%	20.944	20.944	0.19	0.683
Total	8	773.761	100.00%				

**Table 10:** Experimental summary of the wear test.

S. No	% of Reinforcement	Stirring Time	Stirring Speed (rpm)	Wear (µm)	S/N Ratio
1	3	20	500	130.34	−42.3016
2	3	25	550	141.76	−43.0311
3	3	30	600	129.67	−42.2568
4	6	20	550	124.68	−41.9159
5	6	25	600	119.53	−41.5495
6	6	30	500	139.93	−42.9182
7	9	20	600	135.61	−42.6458
8	9	25	500	147.26	−43.3617
9	9	30	550	137.19	−42.7464

**Table 11:** Response table for means (wear).

Level	% of Reinforcement	Stirring Time	Stirring Speed (rpm)
1	133.9	130.2	139.2
2	128.0	136.2	134.5
3	140.0	135.6	128.3
Delta	12.0	6.0	10.9
Rank	1	3	2

**Table 12:** Response table for signal to noise ratios (wear). Smaller is better.

Level	% of Reinforcement	Stirring Time	Stirring Speed (rpm)
1	−42.53	−42.29	−42.86
2	−42.13	−42.65	−42.56
3	−42.92	−42.64	−42.15
Delta	0.79	0.36	0.71
Rank	1	3	2

**Table 13:** Analysis of variance for wear test.

Source	DF	Seq SS	Contribution	Adj SS	Adj MS	F-Value	*p*-Value
Regression	3	277.71	45.35%	277.71	92.57	1.38	0.350
% of reinforcement	1	55.75	9.10%	55.75	55.75	0.83	0.403
Stirring time	1	43.52	7.11%	43.52	43.52	0.65	0.457
Stirring speed (rpm)	1	178.43	29.14%	178.43	178.43	2.67	0.163
Total	8	612.37	100.00%				

**Table 14:** Summary of corrosion test.

S. No	Percentage of Reinforcement	pH Value	Hang Time (Hours)	Corrosion Rate × 10^−3^ (mm/Year)	S/N Ratio
1	3	3	24	0.139	17.1397
2	3	6	48	0.172	15.2894
3	3	9	72	0.196	14.1549
4	6	3	48	0.148	16.5948
5	6	6	72	0.175	15.1392
6	6	9	24	0.131	17.6546
7	9	3	72	0.144	16.8328
8	9	6	24	0.162	15.8097
9	9	9	48	0.183	14.7510

**Table 15:** Response table for means (corrosion test).

Level	Percentage of Reinforcement	pH Value	Hang Time (Hours)
1	0.1690	0.1437	0.1440
2	0.1513	0.1697	0.1677
3	0.1630	0.1700	0.1717
Delta	0.0177	0.0263	0.0277
Rank	3	2	1

**Table 16:** Response table for signal to noise ratios (smaller is better).

Level	Percentage of Reinforcement	pH Value	Hang Time (Hours)
1	15.53	16.86	16.87
2	16.46	15.41	15.55
3	15.80	15.52	15.38
Delta	0.93	1.44	1.49
Rank	3	2	1

**Table 17:** Outline of ANOVA analysis (corrosion test).

Source	DOF	Seq SS	Contribution %	Adj SS	Adj MS	F-Value	*p*-Value
Percentage of reinforcement	1	0.000520	12.36	0.000520	0.000520	1.60	0.067
pH value	1	0.001148	29.68	0.001148	0.001148	2.30	0.119
Hang time (hours)	1	0.002242	57.96	0.002242	0.000747	3.53	0.195
Total	8	0.000007	100.00				

**Table 18:** Experimental summary of microhardness test.

Exp. Runs	% of Reinforcement	pH Value	Hang Time (Hours)	Microhardness (HV)	S/N Ratio
1	3	3	24	138	42.7976
2	3	6	48	144	43.1672
3	3	9	72	136	42.6708
4	6	3	48	152	43.6369
5	6	6	72	155	43.8066
6	6	9	24	148	43.4052
7	9	3	72	134	42.5421
8	9	6	24	150	43.5218
9	9	9	48	142	43.0458

**Table 19:** Response table for means (microhardness test).

Level	% of Reinforcement	pH Value	Hang Time (Hours)
1	139.3	141.3	145.3
2	151.7	149.7	146.0
3	142.0	142.0	141.7
Delta	12.3	8.3	4.3
Rank	1	2	3

**Table 20:** Response table for signal to noise ratios (microhardness test). Larger is better.

Level	% of Reinforcement	pH Value	Hang Time (Hours)
1	42.88	42.99	43.24
2	43.62	43.50	43.28
3	43.04	43.04	43.01
Delta	0.74	0.51	0.28
Rank	1	2	3

**Table 21:** Analysis of variance for microhardness.

Source	DF	Seq SS	Contribution	Adj SS	Adj MS	F-Value	*p*-Value
Regression	3	31.500	7.16%	31.500	10.5000	0.13	0.939
% of reinforcement	1	10.667	2.42%	10.667	10.6667	0.13	0.733
pH Value	1	0.667	0.15%	0.667	0.6667	0.01	0.932
Hang time (hours)	1	20.167	4.58%	20.167	20.1667	0.25	0.640
Total	8	440.000	100.00%				

## Data Availability

Not applicable.

## References

[1] Butola R., Pratap C., Shukla A., Walia R. (2019). Effect on the Mechanical Properties of Aluminum-Based Hybrid Metal Matrix Composite Using Stir Casting Method. Mater. Sci. Forum.

[2] Mahesh V., Joladarashi S., Kulkarni M.S. (2019). Experimental study on abrasive wear behaviour of flexible green composite intended to be used as protective cladding for structures. Int. J. Mod. Manuf. Technol..

[3] Kumar B.P., Birru A.K. (2017). Microstructure and mechanical properties of aluminium metal matrix composites with addition of bamboo leaf ash by stir casting method. Trans. Nonferrous Met. Soc. China.

[4] Pazhouhanfar Y., Eghbali B. (2018). Microstructural characterization and mechanical properties of TiB2 reinforced Al6061 matrix composites produced using stir casting process. Mater. Sci. Eng. A.

[5] Ravi B., Naik B.B., Prakash J.U. (2015). Characterization of Aluminium Matrix Composites (AA6061/B4C) Fabricated by Stir Casting Technique. Mater. Today Proc..

[6] Ezatpour H.R., Sajjadi S.A., Sabzevar M.H., Huang Y. (2013). Investigation of microstructure and mechanical properties of Al6061-nanocomposite fabricated by stir casting. Mater. Des..

[7] Sathish T., Sabarirajan N. (2020). Synthesis and Optimization of AA 7175-Zirconium Carbide composites machining parameters. J. New Mater. Electrochem. Syst..

[8] Chaudhary A., Kumar Dev A., Goel A., Butola R., Ranganath M. (2018). The Mechanical properties of different alloys in friction stir processing: A review. Mater. Today.

[9] Jo M.C., Choi J.H., Yoo J., Lee D., Shin S., Jo I., Lee S.K., Lee S. (2019). Novel dynamic compressive and ballistic properties in 7075–T6 Al-matrix hybrid composite reinforced with SiC and B4C particulates. Compos. B Eng..

[10] Logesh K., Hariharasakthisudhan P., Moshi A.A.M., Rajan B.S., Sathickbasha K. (2019). Mechanical properties and microstructure of A356 alloy reinforced AlN/MWCNT/graphite/Al composites fabricated by stir casting. Mater. Res. Express.

[11] Sathish T., Karthick S. (2020). Wear behaviour analysis on aluminium alloy 7050 with reinforced SiC through taguchi approach. J. Mater. Res. Technol..

[12] Akhlaghi F., Zare-Bidaki A. (2009). Influence of graphite content on the dry sliding and oil impregnated sliding wear behavior of Al 2024—Graphite composites produced by in situ powder metallurgy method. Wear.

[13] Idrisi A.H., Mourad A.-H.I. (2017). Fabrication and wear analysis of aluminium matrix composite reinforced by sic micro and nano particles. Proceedings of the ASME 2017 Pressure Vessels and Piping Conference.

[14] Esakkiraj E.S., Suresh S., Moorthi N., Kumar M.K., Ranjith S.M., Diaz P.M., Palanikumar K., Kumar P.R. (2014). Study of mechanical behaviour of stir cast aluminium based composite reinforced with mechanically ball milled TiB2 nano particles. Advanced Materials Research.

[15] Girish B., Satish B., Sarapure S., Somashekar D., Basawaraj S. (2015). Wear behavior of magnesium alloy AZ91 hybrid composite materials. Tribol. Trans..

[16] Sathish T., Arunkumar S., Saravanan R., Dhinakaran V. (2020). Experimental investigation on material characterization of zirconia reinforced Alumina ceramic composites via powder forming process. AIP Conference Proceedings.

[17] Chourasiya S.K., Gautam G., Singh D. (2020). Mechanical and tribological behavior of warm rolled Al-6Si-3graphite self lubricating composite synthesized by spray forming process. Silicon.

[18] Prasad R.A., Vamsi K.P., Rao R.N. (2019). Tribological behaviour of Al6061–2SiC-xGr hybrid metal matrix nanocomposites fabricated through ultrasonically assisted stir casting technique. Silicon.

[19] Kaushik N., Rao R.N. (2016). The effect of wear parameters and heat treatment on two body abrasive wear of Al-SiC-Gr hybrid composites. Tribol. Int..

[20] Wang C., Deng K., Bai Y. (2019). Microstructure, and mechanical and Wear properties of Grp/AZ91 magnesium matrix composites. Materials.

[21] Sathish T., Muthu G., Vijayakumar M.D., Dhinakaran V., Bupathi Ram P.M. (2020). Mechanical Properties and Microstructural analysis of Friction stir Processed AA6056-Zirconium dioxide (ZrO2). Mater. Today Proc..

[22] Zhu J., Qi J., Guan Q., Ma L., Joyce R. (2020). Tribological behaviour of self-lubricating Mg matrix composites reinforced with silicon carbide and tungsten disulfide. Tribol. Int..

[23] Stojanovic B., Babic M., Velickovic S., Blagojevic J., Velickovic S. (2016). Tribological behavior of aluminum hybrid composites studied by application of factorial techniques. Tribol. Trans..

[24] Senthilkumar M., Saravanan S., Shankar S. (2015). Dry sliding wear and friction behavior of aluminum–rice husk ash composite using Taguchi’s technique. J. Compos. Mater..

[25] Aravindan S., Rao P.V., Ponappa K. (2015). Evaluation of physical and mechanical properties of AAZ91D/SiC composites by two step stir casting process. J. Magnes. Alloy..

[26] Verma R., Suri N.M., Kant S. (2019). Process optimization of slurry spray technique through multi-attribute utility function. Arab. J. Sci. Eng..

[27] Lara R.D., Soltani N., Bahrami A., Castaneda E.G., Sanchez E.G., Rodriguez M.A.L.H. (2015). Tribological characterization of Al7075–graphite composites fabricated by mechanical alloying and hot extrusion. Mater. Des..

[28] Shen Q., Wu C., Luo G., Fang P., Li C., Wang Y., Zhang L. (2014). Microstructure and mechanical properties of Al-7075/B4C composites fabricated by plasma activated sintering. J. Alloys Compd..

[29] Baradeswaran A., Vettivel S.C., ElayaPerumal A., Selvakumar N., Franklin Issac R. (2014). Experimental investigation on mechanical behaviour, modeling and optimization of wear parameters of B4C and graphite reinforced aluminium hybrid composites. Mater. Des..

[30] Alaneme K.K., Sanusi K.O. (2015). Microstructural characteristics, mechanical and wear behaviour of aluminium matrix hybrid composites reinforced with alumina, rice husk ash, and graphite. Eng. Sci. Technol. Int. J..

[31] Chen W., Liu Y., Yang C., Zhu D., Li Y. (2014). (SiCp + Ti)/7075 Al hybrid composites with high strength and great plasticity fabricated by squeeze casting. Mater. Sci. Eng. A.

[32] Fale S., Likhite A., Bhatt J. (2014). Compressive, Tensile and Wear Behavior of Ex Situ Al/AlN Metal Matrix Nanocomposites. J. Compos. Mater..

[33] Pourhosseini S., Beygi H., Sajjadi S.A. (2018). Effect of Metal Coating of Reinforcements on the Microstructure and Mechanical Properties of Al-Al_2_O_3_ Nanocomposites. Mater. Sci. Technol..

[34] Tian W.S., Zhao Q.L., Zhao C.J., Qiu F., Jiang Q.C. (2017). The Dry Sliding Wear Properties of Nano-Sized TiCp/Al-Cu Composites at Elevated Temperatures. Materials.

[35] Kmec J., Fechova E., Hrehova S. (2018). Optimization of parameters of the plastic-coated sheets at the corrosion test in salt spray. MM Sci. J..

[36] Prabhuraj P., Rajakumar S., Balasubramanian V. (2018). Optimising salt fog corrosion parameters to minimizing the corrosion rate of AA7075-T651 alloy. Mater. Today Proc..

[37] Stoll M., Stemmer F., Ilinzeer S., Weidenmann K.A. (2017). Optimization of Corrosive Properties of Carbon Fiber Reinforced Aluminum Laminates due to Integration of an Elastomer Interlayer. Key Eng. Mater..

[38] Sathish T., Sevvel P., Sudharsan P., Vijayan V. (2020). Investigation and optimization of laser welding process parameters for AA7068 aluminium alloy butt joint. Mater. Today Proc..

[39] Anitha C., Azim S.S., Mayavan S. (2018). Influence of particle size in fluorine free corrosion resistance superhydrophobic coating—Optimization and stabilization of interface by multiscale roughness. J. Alloys Compd..

[40] Abd El-Aziz K., Saber D., Sallam H.E.D.M. (2015). Wear and Corrosion Behavior of Al–Si Matrix Composite Reinforced with Alumina. J. Bio-Tribo-Corros..

[41] Sathish T. (2021). Nano-Alumina Reinforcement on AA 8079 acquired from Waste Aluminium Food Containers for altering Microhardness and Wear resistance. J. Mater. Res. Technol..

[42] Meignanamoorthy M., Ravichandran M., Mohanavel V., Afzal A., Sathish T., Alamri S., Khan S., Saleel C. (2021). Microstructure, Mechanical Properties, and Corrosion Behavior of Boron Carbide Reinforced Aluminum Alloy (Al-Fe-Si-Zn-Cu) Matrix Composites Produced via Powder Metallurgy Route. Materials.

[43] Mysore T., Patil A., Raju G., Banapurmath N., Bhovi P., Afzal A., Alamri S., Saleel C. (2021). Investigation of Mechanical and Physical Properties of Big Sheep Horn as an Alternative Biomaterial for Structural Applications. Materials.

[44] Sathish T., Mohanavel V., Ansari K., Saravanan R., Karthick A., Afzal A., Alamri S., Saleel C. (2021). Synthesis and Characterization of Mechanical Properties and Wire Cut EDM Process Parameters Analysis in AZ61 Magnesium Alloy + B_4_C + SiC. Materials.

[45] Sharath B., Venkatesh C., Afzal A., Aslfattahi N., Aabid A., Baig M., Saleh B. (2021). Multi Ceramic Particles Inclusion in the Aluminium Matrix and Wear Characterization through Experimental and Response Surface-Artificial Neural Networks. Materials.

[46] Sathish T., Kaladgi A., Mohanavel V., Arul K., Afzal A., Aabid A., Baig M., Saleh B. (2021). Experimental Investigation of the Friction Stir Weldability of AA8006 with Zirconia Particle Reinforcement and Optimized Process Parameters. Materials.

[47] Akhtar M., Khan M., Khan S., Afzal A., Subbiah R., Ahmad S., Husain M., Butt M., Othman A., Bakar E. (2021). Determination of Non-Recrystallization Temperature for Niobium Microalloyed Steel. Materials.

[48] Nagaraja S., Nagegowda K.U., Kumar V.A., Alamri S., Afzal A., Thakur D., Kaladgi A.R., Panchal S., Saleel C. (2021). AInfluence of the Fly Ash Material Inoculants on the Tensile and Impact Characteristics of the Aluminum AA 5083/7.5SiC Composites. Materials.

[49] Akhtar M., Sathish T., Mohanavel V., Afzal A., Arul K., Ravichandran M., Rahim I., Alhady S., Bakar E., Saleh B. (2021). Optimization of Process Parameters in CNC Turning of Aluminum 7075 Alloy Using L27 Array-Based Taguchi Method. Materials.

